# Biomarkers of Preclinical Diabetic Retinopathy Detected by OCT Angiography—A Descriptive Review

**DOI:** 10.3390/life16030496

**Published:** 2026-03-18

**Authors:** Ilona Strauss, Maciej Gawęcki

**Affiliations:** Dobry Wzrok Ophthalmological Center, 80-392 Gdansk, Poland; ilonastrauss@wp.pl

**Keywords:** preclinical diabetic retinopathy, OCTA, biomarkers, vessel density, perfusion density, FAZ

## Abstract

**Background**: Diabetic retinopathy (DR) is a leading cause of vision loss worldwide. Microvascular changes precede clinically detectable DR, creating an opportunity for early diagnosis and intervention. Optical coherence tomography angiography (OCTA) enables noninvasive, quantitative assessments of retinal and choroidal microcirculation and has emerged as a promising tool for identifying early biomarkers of DR. The goal of this study was to review the literature on OCTA-derived biomarkers associated with preclinical diabetic retinopathy in patients with type 1 and type 2 diabetes mellitus. **Methods**: This descriptive literature review summarizes current evidence regarding OCTA-derived biomarkers associated with preclinical diabetic retinopathy in patients with type 1 and type 2 diabetes mellitus. A search of the PubMed/MEDLINE database was performed to identify original studies published between 2015 and 2025 evaluating OCTA parameters in diabetic patients without clinically detectable diabetic retinopathy. The findings were synthesized qualitatively due to methodological heterogeneity among studies in terms of OCTA devices, imaging protocols, and analyzed parameters. **Results**: The reviewed studies consistently reported early microvascular abnormalities detectable by OCTA prior to the development of clinically visible diabetic retinopathy. The most frequently described changes included reduced vessel density (VD) and perfusion parameters, enlargement and increased irregularity of the foveal avascular zone (FAZ), areas of capillary non-perfusion, and alterations in vascular network geometry and complexity. These changes were most consistently observed in the deep capillary plexus (DCP), suggesting that this vascular layer may be particularly susceptible to early diabetic microvascular damage. **Conclusions**: This review provides a comprehensive synthesis of OCTA-derived biomarkers associated with early retinal microvascular alterations in diabetic patients without clinically detectable diabetic retinopathy. By integrating findings from recent studies, the review highlights the potential role of OCTA in identifying preclinical microvascular changes and discusses current methodological challenges and future research directions.

## 1. Introduction

Diabetes mellitus is a global metabolic disease with increasing prevalence, constituting one of the main causes of morbidity and premature mortality worldwide. In recent decades, a continuous increase in the number of diabetes cases has been observed, which is associated with the epidemic of obesity, population aging, urbanization, and lifestyle changes [1]. According to the latest 11th edition of the IDF Diabetes Atlas 2025 published by the International Diabetes Federation (IDF), in 2024 approximately 589 million adults (aged 20–79 years) were living with diabetes worldwide, corresponding to about 11.1% of the global adult population.

Type 2 diabetes mellitus (T2DM) accounts for the vast majority of diabetes cases worldwide and is strongly associated with obesity, sedentary lifestyle, and population aging [1,2,3]. In contrast, type 1 diabetes mellitus (T1DM) typically develops at a younger age and results from the autoimmune destruction of pancreatic β-cells, leading to absolute insulin deficiency. Because patients with T1DM are exposed to chronic hyperglycemia for a longer period of time, this population may represent a valuable clinical model for studying early microvascular changes in diabetes. In T2DM, retinal microvascular alterations may additionally reflect the influence of systemic comorbidities such as hypertension, dyslipidemia, and obesity. These differences should be considered when interpreting OCTA-derived biomarkers.

Diabetic retinopathy (DR) is one of the most common microvascular complications of diabetes and remains a major cause of vision loss among working-age adults [4,5]. Importantly, microvascular and neurodegenerative retinal changes may develop before clinically visible lesions are detected during routine fundus examination or color fundus photography. This stage is commonly referred to as preclinical diabetic retinopathy, defined as the phase in which no clinically detectable signs of DR are present despite the presence of subtle retinal abnormalities that may be identified using more sensitive imaging modalities, such as recently introduced optical coherence tomography angiography (OCTA).

### OCT Angiography (OCTA)

Traditionally, diabetic retinopathy was diagnosed using fluorescein angiography (FA) and, subsequently, optical coherence tomography (OCT). For many years, FA has been considered the gold standard for evaluating retinal vascular abnormalities in diabetic retinopathy. It provides dynamic information about vascular leakage and perfusion; however, it is invasive, requires intravenous dye administration, and may be associated with adverse reactions. In contrast, OCTA is a noninvasive technique that enables the rapid visualization of retinal capillary networks and a quantitative assessment of microvascular parameters such as vessel density and the foveal avascular zone.

OCT has become a fundamental imaging modality in modern ophthalmology, enabling a noninvasive, high-resolution cross-sectional visualization of retinal structures. OCTA was introduced as an extension of OCT technology, allowing depth-resolved visualization of retinal and choroidal microvasculature without the need for dye injection.

Despite these advantages, OCTA also has important limitations. Unlike FA, OCTA does not directly visualize vascular leakage and may be affected by image artifacts, segmentation errors, and variability between devices. Therefore, OCTA should be considered a complementary imaging modality that provides detailed structural and microvascular information but does not fully replace conventional angiographic techniques.

The goal of this review was to evaluate OCTA biomarkers used for the detection and assessment of preclinical diabetic retinopathy, with particular emphasis on their clinical relevance and usefulness in ophthalmological practice

## 2. Materials and Methods

**Figure 1:**
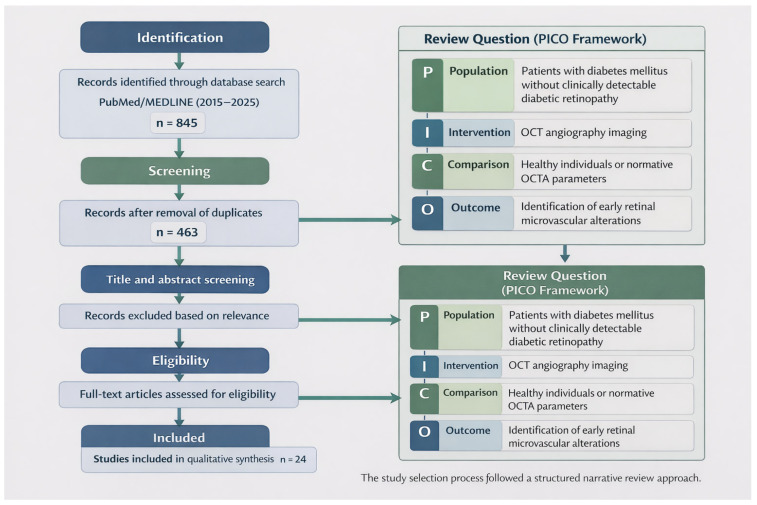
Study selection process flow-chart.

A literature search was performed in the PubMed/MEDLINE database to identify studies investigating OCTA-derived biomarkers in patients with diabetes without clinically apparent diabetic retinopathy. The search strategy combined terms related to optical coherence tomography angiography (e.g., “OCTA”, “optical coherence tomography angiography”) and diabetic retinal disease (e.g., “diabetic retinopathy”, “early diabetic retinal changes”, “preclinical diabetic retinopathy”, and “microvascular alterations”). In addition, the reference lists of eligible articles were manually screened to identify further relevant publications. The flow-chart of the study selection process is illustrated in Figure 1.

Original research articles were included if they met the following criteria:

(1) Evaluation of patients with type 1 or type 2 diabetes mellitus;
(2) Inclusion of patients without clinically apparent diabetic retinopathy or explicitly defined preclinical/no-DR stages;
(3) Use of OCTA to assess retinal and/or choroidal microcirculation;
(4) Reporting of quantitative OCTA parameters, such as vessel density, perfusion density, foveal avascular zone (FAZ) metrics, ischemic indices, or choriocapillaris flow parameters;
(5) Inclusion of adult and/or pediatric populations.

Review articles, case reports, conference abstracts, animal studies, and studies without quantitative OCTA analysis were excluded.

Given the methodological heterogeneity across published studies, including differences in OCTA devices, image processing algorithms, definitions of preclinical diabetic retinopathy, and reported vascular parameters, a descriptive review design was chosen. This approach allows a qualitative synthesis of the existing evidence and facilitates discussion of emerging OCTA biomarkers and their potential role in the early detection of diabetic retinal microvascular changes.

It should be noted that the definition of preclinical or no-diabetic-retinopathy (no-DR) status varied across the included studies. In most cases, this stage was defined as the absence of clinically detectable diabetic retinopathy based on fundus examination or color fundus photography, although the exact grading protocols and inclusion criteria differed between studies. Because of this variability, the present review focuses on identifying general trends in OCTA-derived biomarkers rather than performing direct quantitative comparisons between individual studies.

Because this work was designed as a narrative review and the included studies showed substantial methodological heterogeneity in imaging protocols, OCTA devices, and reported biomarkers, a formal risk-of-bias assessment was not performed. Therefore, the findings of this review should be interpreted with caution.

As this study was based exclusively on previously published data, no institutional review board approval or informed consent was required.

## 3. Results

Following our search in all databases, 845 articles were identified. After the removal of duplicates and evaluation of the titles and abstracts, 463 remained. Finally, based on the inclusion criteria and assessment of full text articles, 24 articles that were published in the years 2015–2025 were included in the study.

After an evaluation of the most frequently described OCTA parameters in diabetic retinopathy, the review concentrated on the following categories: vessel density and retinal perfusion, foveal avascular zone, ischemia and areas of non-perfusion, microaneurysms, blood flow parameters, retinal layer thickness, choriocapillaris and choroid, parameters of vascular geometry, and network complexity and other factors.

Most authors described changes in the retina in patients with both early and advanced diabetic retinopathy, but few authors included changes in patients with preclinical diabetic retinopathy in their studies.

Most publications were based on a small research group focusing mainly on T2DM. Among all the publications analyzed, only a few authors described groups of more than 100 patients [6,7,8,9].

A significant limitation of the available data was the lack of longitudinal studies assessing the dynamics of microcirculatory changes in patients with long-standing T1DM without DR. Most publications concerned patients with type 2 diabetes or included short follow-up periods [4,5,8,9]. Meanwhile, T1DM, due to the long duration of the disease and often the absence of other comorbidities, such as hypertension, is a unique model for analyzing early, subclinical retinal changes [4,10].

### 3.1. Most Frequently Described OCTA Parameters in Patients with Diabetes Mellitus

The development of optical coherence tomography angiography (OCTA) has enabled noninvasive, quantitative assessments of retinal and choroidal microcirculation in patients with diabetes mellitus, including at the very early stages of the disease, even before the appearance of clinical signs of diabetic retinopathy [11,12,13,14,15] (Table 1). In recent years, numerous cross-sectional and longitudinal studies have demonstrated that OCTA reveals subtle disturbances in perfusion and capillary network architecture in patients with type 1 and type 2 diabetes mellitus, even in groups without clinically diagnosed DR [6,10,11,12,13,14,15,16,17,18,19,20,21,22,23,24,25,26].

### 3.2. Vessel Density and Retinal Perfusion

The most frequently analyzed OCTA parameter in diabetes mellitus is vessel density (VD) and related perfusion indices [4,5,6,7,8,9,10,15,16,17,21,22,27,28,29,30,31,32,33,40,41,42]. Numerous studies have demonstrated a significant reduction in macular VD in patients with diabetes compared with healthy individuals [4,5,9,10,16,21,22,28,29,30,41,42].

Importantly, many studies indicate that these disturbances primarily affect the deep capillary plexus (DCP), while the superficial capillary plexus (SCP) initially remains relatively preserved [4,9,10,16,17,18,29,30,40,41]. This selective reduction of VD in the DCP has been observed in both adults and children or adolescents with diabetes without clinical signs of DR, suggesting that the DCP is the most vulnerable vascular plexus to early diabetic damage [6,9,10,16].

It is believed that deeper retinal vessels are more susceptible to damage in the course of diabetes mellitus. Numerous studies have demonstrated a negative correlation between the duration of diabetes and VD values in the DCP [9,10,16,29,40,41,42].

The deep capillary plexus appears particularly vulnerable in early diabetic microangiopathy due to several structural and physiological factors. The DCP is located within deeper layers of the inner retina, where oxygen tension is relatively lower and metabolic demand is high, making this region more susceptible to hypoxic injury and metabolic stress. In early diabetes, microvascular dysfunction develops, including endothelial damage, impaired autoregulation, and increased capillary pressure. These alterations may disproportionately affect the DCP because of its anatomical position and relatively limited collateral circulation, which reduces the ability of this vascular layer to compensate for perfusion disturbances [34,35].

Several studies indicate that microvascular alterations within the deep retinal vascular layer may occur earlier in the course of diabetes [4,9,10,16,29,40,41]. Most investigations suggest that the DCP is particularly vulnerable to early diabetic damage. However, early alterations in the SCP have also been reported in patients without clinically detectable diabetic retinopathy [4,5,6,7,9]. With disease progression and the development of clinically manifest DR, further reductions in vessel density are observed in the SCP, and the magnitude of VD reduction in both plexuses correlates with the severity of clinical disease [28,29,30,32,41,42]. Longitudinal studies have demonstrated that a decrease in vessel density, particularly in the DCP, may precede the onset of clinically detectable diabetic retinopathy and may therefore serve as a potential prognostic biomarker.

Patients with diabetic macular edema (DME) exhibited significantly lower VD values in the DCP compared with patients with DR without DME, and a negative correlation was observed between vessel density across the entire SCP area and visual acuity [28,29,30,32,41]. A reduction in VD in the DCP may represent an earlier marker of DR in patients with diabetes mellitus than FAZ enlargement [4,9,10,25,40].

#### Clinical Relevance for Preclinical DR

A reduction in VD in the DCP, even when values remain close to the lower limit of normal, represents one of the earliest and most reproducible markers of preclinical diabetic retinopathy.

### 3.3. Foveal Avascular Zone

An important area of OCTA analysis is the foveal avascular zone. Numerous publications have demonstrated an enlargement of the FAZ area and increased FAZ irregularity in patients with diabetes mellitus, including groups without clinical DR [4,9,17,31,33]. Beyond FAZ area alone, increasing attention has been given to FAZ shape indices, such as perimeter, circularity index, and acircularity index, which appear to be more sensitive to early microvascular changes than simple FAZ area measurement. Some authors have reported FAZ enlargement in both the SCP and DCP in patients with diabetes without DR [4,9,17,31]. There are also many studies describing no significant differences in FAZ parameters in patients with diabetes without DR compared with control groups [6,7,10], as well as FAZ enlargement in the DCP in patients with diabetes across all disease stages [4,9,17,31].

Inconsistencies exist in the literature regarding the diagnostic utility and specificity of foveal avascular zone (FAZ) enlargement as a biomarker of preclinical diabetic retinopathy. The FAZ area exhibits substantial inter-individual variability in healthy eyes [36], resulting in a considerable overlap between diabetic and non-diabetic populations and thereby limiting its discriminatory value. Some studies report significant FAZ enlargement in patients with prediabetes or diabetes without clinically detectable retinopathy [37,38], whereas others describe only modest or non-significant differences, particularly in the deep capillary plexus. These discrepancies may also be explained by methodological factors, including differences in OCTA devices, scan sizes, segmentation algorithms, and FAZ measurement protocols.

A positive correlation between FAZ size and best-corrected visual acuity (BCVA) has been demonstrated [28,30,32], particularly pronounced in patients with diabetic macular edema [29,30,41].

Manual FAZ segmentation is characterized by high variability [43], and segmentation errors have represented a limitation to its use as a biomarker [44,45]. Some authors have proposed the use of automated algorithms for FAZ delineation and diagnostic evaluation [43,45,46].

In healthy individuals, the FAZ is circular or elliptical in shape. Its area measured by OCTA averages in the range 0.25–0.35 mm^2^ in the SCP and 0.40–0.60 mm^2^ in the DCP, with significant variability depending on the OCTA device and segmentation algorithm used [17,34,45].

The FAZ area increases with age and is also larger in individuals with myopia or arterial hypertension [17,34,45]. A reduction in vessel density in the immediate vicinity of the FAZ (peri-FAZ vessel density) has been described as one of the earliest markers of macular ischemia and often precedes a clear enlargement of the avascular zone itself.

### 3.4. Ischemia and Areas of Non-Perfusion

Another important group of OCTA biomarkers comprises indicators of retinal ischemia, including areas of capillary non-perfusion, capillary dropout or closure, and the presence of flow voids. Studies demonstrate that these changes appear early—particularly within the deep capillary plexus—and increase in extent with the progression of diabetic retinopathy [4,9,10,28,29,30,41,42].

Widefield OCTA analyses have shown that the extent of ischemia beyond the macular region correlates with the risk of progression to advanced stages of diabetic retinopathy, including proliferative diabetic retinopathy. Parameters such as the ischemic index are increasingly proposed as tools for clinical risk stratification.

### 3.5. Microaneurysms and Vascular Activity

OCTA also enables the visualization of microaneurysms, including their number, location, and the presence of flow within their lumen. Although microaneurysms are classically associated with fluorescein angiography, multiple studies have demonstrated that OCTA allows the assessment of their morphology and hemodynamic activity, particularly within the deep vascular plexuses. The presence of a flow signal within microaneurysms is interpreted as a marker of disease activity and may have prognostic significance [29,32,47]. Microaneurysms have been recognized as one of the most useful indicators in international diabetic retinopathy severity scales [48].

OCTA studies have shown that microaneurysms are distributed across all retinal vascular plexuses, although the majority are visible in the DCP and intermediate capillary plexus (ICP). Histopathological studies localize microaneurysms primarily within the inner nuclear layer (INL), which contains the lower portion of the ICP and the upper portion of the DCP. Dubow et al. classified microaneurysms into saccular, fusiform, mixed, pedunculated, irregular, and focal bulging types. Irregular, fusiform, and mixed microaneurysms demonstrated the greatest tendency toward leakage [47,49].

### 3.6. Blood Flow Parameters

Reduced retinal blood flow has been demonstrated in both humans and animal models with early diabetes mellitus. This phenomenon has been linked to increased diacylglycerol (DAG) levels and activation of protein kinase C-β isoforms [50]. As diabetic retinopathy progresses, blood flow increases, suggesting that initial flow resistance occurs at the level of retinal arterioles and capillaries, potentially due to increased endothelin-1 expression and elevated VEGF production secondary to hypoxia [50]. Flow values are measured as the percentage of pixels with flow signal relative to the total number of pixels in the analyzed area [11]. Studies have demonstrated a reduction of this parameter in all examined SCP and DCP regions [28,29,30,41,42]. Flow parameters within the inferior macular sector have been shown to exhibit greater sensitivity in identifying high-risk groups. This is attributed to the fact that this region undergoes embryological closure around the 34th week of gestation and consists of small ganglion cell axons [28,29,41].

### 3.7. Retinal Layer Thickness

In preclinical and mild stages of diabetic retinopathy, a reduction in retinal layer thickness has been observed [6,10,16], whereas in moderate stages of DR, retinal thickness increases [28,29,41]. Some studies have reported no significant differences in retinal thickness between patients with diabetes without DR and healthy control subjects [6,7]. Other studies have demonstrated a significant reduction in ganglion cell–inner plexiform layer (GCIPL) thickness in the outer macular region in patients with diabetes without DR, and in the inner macular region in patients with mild DR. Thinning of the GCIPL in patients with diabetes without DR has been attributed mainly to ganglion cell layer (GCL) thinning, whereas changes observed in mild DR were associated with thinning of the inner plexiform layer (IPL).

The ganglion cell layer consists predominantly of retinal ganglion cell bodies and represents a critical component of the inner retina responsible for visual signal transmission. Increasing evidence suggests that neurodegenerative processes may occur in the diabetic retina before the onset of clinically detectable microvascular abnormalities. Structural alterations in the inner retinal layers, including thinning of the ganglion cell layer and ganglion cell–inner plexiform layer complex, have been reported in diabetic patients without clinically apparent diabetic retinopathy, supporting the concept that retinal neurodegeneration may precede the classical vascular manifestations of the disease [51].

More broadly, mechanisms of retinal ganglion cell degeneration have also been investigated in other retinal diseases, including glaucoma, highlighting the vulnerability of inner retinal neurons to metabolic and cellular stress [52].

These findings highlight the potential value of advanced retinal imaging techniques, such as OCT and OCTA, for detecting early neurovascular alterations in diabetes.

The IPL contains numerous synapses connecting dendrites with bipolar and amacrine cells and is affected later, when retinopathy becomes clinically apparent [28,29,41].

In eyes with moderate DR, increased retinal thickness has been observed across all macular regions, involving the full thickness of the macula. Layer-specific analysis revealed that thickening occurs mainly in the INL and outer nuclear layer (ONL), with an increase in GCIPL thickness—particularly in the IPL [28,29,41]. Moderate DR is associated with the presence of microaneurysms, more than 80% of which are located within the INL [47]. It is also suggested that thickening of these layers may result from cytotoxic neuronal edema secondary to ischemia, in the absence of visible vascular leakage [50]. The presence of vascular leakage triggers an inflammatory response, including infiltration by inflammatory cells (microglia), which may further increase edema [50].

Some studies did not demonstrate changes in ONL and INL thickness in patients with non-proliferative diabetic retinopathy (NPDR) without DME; however, these studies were based on small patient cohorts. Seven-year longitudinal studies confirmed an increase in inner retinal thickness from the inner limiting membrane (ILM) to the outer plexiform layer (OPL) in patients with type 2 diabetes mellitus without DME [42]. Other authors reported a 4.1% increase in IPL-ONL complex thickness in patients with type 1 diabetes mellitus compared with control subjects [6].

### 3.8. Choriocapillaris and Choroid

An increasing number of studies focus on the assessment of the choroid and choriocapillaris using OCTA. In patients with diabetes mellitus, including those without clinically evident diabetic retinopathy, an increased proportion of areas with choriocapillaris flow deficit has been described, suggesting that choroidal involvement may occur very early in the disease course. In patients with diabetes, the presence of microaneurysms, vascular dilations, as well as vessel closure or structural modifications such as increased tortuosity, has been observed in the choroid [15,21,22].

Longitudinal studies have demonstrated that choriocapillaris perfusion disturbances may be associated with an increased risk of developing diabetic retinopathy requiring treatment as well as diabetic macular edema. In patients with type 1 diabetes mellitus without diabetic retinopathy, thinning of choroidal thickness has been reported [6,53]. Some studies found no changes in choriocapillaris vessel density in patients with diabetes without DR, while a decrease in choriocapillaris vessel density was observed with DR progression [15,21]. Other studies have shown that during a one-year follow-up of patients with diabetes without DR, an increase in choroidal thickness was observed, which was attributed to the presence of choroidal vasculopathy [53]. Choriocapillaris vessel density was significantly associated with diastolic blood pressure in the group of patients with diabetes without DR [15].

### 3.9. Parameters of Vascular Geometry and Network Complexity

In addition to classical measures of vessel density and perfusion, the literature increasingly analyzes parameters describing vascular network geometry, such as vessel tortuosity, vascular length density (VLD) [22,30], fractal dimension (FD) [39], lacunarity, and vascular complexity indices, including measures of sphericity and cylindricity of vessels [36]. Fractal dimension is a quantitative parameter describing the geometric complexity of the retinal vascular network, whereas lacunarity reflects the spatial heterogeneity and distribution of gaps within that network.

Studies indicate that a reduction in topological complexity of the capillary network and an increase in its heterogeneity may represent very early markers of subclinical diabetic microangiopathy [30,39]. A decrease in FD, parafoveal vessel density (PVD), and parafoveal vessel length density (PVLD) in the superficial capillary plexus, along with an increase in blood vessel tortuosity (BVT), was significantly associated with an increased risk of diabetic retinopathy. A reduction in FD was found to be more significant than increased BVT [39]. Fractal dimension may represent a highly valuable quantitative parameter for assessing microvascular damage across different stages of diabetic retinopathy [39]. Li et al. demonstrated a reduction in VLD in patients with diabetes mellitus without diabetic retinopathy [19].

### 3.10. Other OCTA-Derived Parameters

An interesting parameter described by Ragkousis et al. is FD-300, defined as vessel density measured within 300 µm from the foveal center. This parameter was shown to be the most discriminative for detecting early changes [8].

Viggiano et al. reported a reduction in perfusion density (PD) in both the superficial and deep capillary plexuses in patients with non-proliferative diabetic retinopathy compared with healthy individuals [24]. The greatest reduction was observed in the temporal sector. Earlier studies also identified the temporal region as the most sensitive area for predicting mild NPDR [28,29,41].

In prospective studies, Sun et al. demonstrated that FAZ parameters, vessel density, and flow rate (FR) in the deep capillary plexus may predict disease progression, whereas vessel density in the superficial capillary plexus was associated with the development of diabetic macular edema [54].

The impact of type 1 diabetes mellitus on large systemic vessels has also been investigated, focusing on the carotid artery and the presence of microvascular retinal changes. A reduction in mean vessel density values in both the SCP and DCP was observed in patients with type 1 diabetes compared with control subjects, along with an increase in mean thickness of the middle and inner carotid artery wall as a marker of diabetic macroangiopathy [6].

Correlations have also been described between diabetic retinopathy-type changes and serum levels of brain-derived neurotrophic factor (BDNF), which may serve as a marker distinguishing patients with diabetic maculopathy from those without, as well as patients with diabetic retinopathy from those without retinopathy [55,56].

#### 3.10.1. Preclinical DR in T1DM—Clinical Data

As presented in Table 2, the majority of OCTA studies in patients with T1DM without diabetic retinopathy demonstrated measurable microcirculatory abnormalities compared with healthy individuals. These changes were observed regardless of patient age and included both adult patients with T1DM and children and adolescents. The presence of these abnormalities in pediatric patients emphasizes that microangiopathic processes may begin very early in the disease course, even in the absence of any clinical symptoms.

#### 3.10.2. Pediatric Populations—Clinical Significance

Table 2 clearly distinguishes studies involving children and adolescents with T1DM. In this group, reduced vessel density—particularly in the deep capillary plexus—as well as FAZ alterations were also observed compared with control groups. This is of particular clinical relevance because it involves patients with short disease duration and no clinical signs of retinopathy.

In children with type 1 diabetes mellitus without clinical diabetic retinopathy, OCTA studies consistently demonstrate reduced vessel density in both the superficial and deep capillary plexuses, particularly in the parafoveal and peripapillary regions, compared with healthy controls [6,19]. These changes are typically subtle and may precede the development of clinically detectable retinopathy [19]. Parameters such as the foveal avascular zone area, FAZ perimeter, acircularity index, and foveal vessel density have been shown to be sensitive indicators of early microvascular alterations in pediatric populations [6,18].

In adults and young adults with T1DM, similar OCTA-derived metrics are evaluated; however, microvascular abnormalities are often more pronounced. Studies frequently report greater reductions in parafoveal vessel density, larger FAZ area, and more marked alterations in foveal vascular density [18]. Moreover, the association between higher HbA1c levels and microvascular impairment appears stronger in adults [18], and early onset of diabetes may be associated with more severe alterations in vessel density and FAZ-related parameters. In addition, volumetric OCTA imaging may reveal perfusion defects in the deep capillary plexus before clinically detectable retinopathy in adults, which may be less apparent in conventional two-dimensional scans.

#### 3.10.3. Importance of Methodology and Normative Data

A comparison of preclinical T1DM study results with normative data highlights the crucial role of methodological standardization in OCTA interpretation. As shown in normative studies, FAZ and vessel density values depend on age, OCTA system, scan size, and segmentation algorithms. Therefore, even small deviations from normal ranges—particularly in the deep capillary plexus—may be clinically meaningful in the context of preclinical diabetic retinopathy when interpreted with these factors in mind.

The combined analysis of the data presented in Table 2 indicates that patients with T1DM without clinical signs of diabetic retinopathy exhibit early, measurable disturbances in retinal microcirculation. These changes most commonly include reduced vessel density in the deep capillary plexus and alterations in FAZ parameters. The consistency of these observations across adult and pediatric populations confirms that preclinical DR in T1DM represents a significant and clinically relevant disease stage, in which OCTA may play a key role in assessing the risk of further progression.

Below are studies exclusively involving diabetes mellitus without clinical signs of diabetic retinopathy (preclinical/no DR).

#### 3.10.4. Conclusion (Preclinical DR)

The earliest changes involve reduced vessel density (VD), particularly in the deep capillary plexus, and FAZ enlargement. These changes occur before the development of clinically visible diabetic retinopathy in both T1DM and T2DM.

#### 3.10.5. Limitations

Despite the growing number of publications, the interpretation of OCTA findings in T1DM remains challenging.

Significant heterogeneity exists regarding OCTA devices, acquisition protocols, scan sizes, and segmentation algorithms. Additionally, manual or semi-automated delineations of FAZ and vascular plexus boundaries introduce observer-dependent variability.

Segmentation sensitivity represents an additional important source of variability in OCTA analysis. Even small shifts in the boundary between the superficial and deep vascular plexuses may substantially influence quantitative measurements; for example, FAZ area may change by up to approximately 15% per 10 μm displacement of the segmentation boundary. This issue is particularly relevant for the analysis of the deep capillary plexus (DCP), where segmentation errors and projection artifacts may affect the accuracy of OCTA-derived parameters [58].

Several limitations of the currently available literature should be acknowledged. First, substantial methodological and clinical heterogeneity exists among studies investigating OCTA-derived biomarkers in diabetic patients without clinically detectable retinopathy. Differences in the definition of preclinical or no-diabetic-retinopathy stages, imaging protocols, OCTA devices, segmentation algorithms, and manual or semi-automated delineations of FAZ and vascular plexus may contribute to variability in reported microvascular parameters, such as vessel density or foveal avascular zone metrics [59].

Additionally, different OCTA platforms (e.g., Optovue (Fremont, CA, USA), Zeiss (Jena, Germany), and Topcon (Tokyo, Japan))) employ distinct hardware configurations, scanning protocols, segmentation algorithms, and proprietary image-processing methods, which may lead to variability in quantitative measurements such as vessel density or the foveal avascular zone (FAZ) area. Consequently, FAZ measurements may vary significantly between OCTA devices, and the obtained values are not always directly interchangeable across studies. These differences are both device- and layer-specific and may substantially influence the interpretation of OCTA-derived metrics. Therefore, standardization of acquisition and analysis protocols is essential for reliable comparisons in longitudinal and multicenter studies [60,61].

In addition, the studied populations vary considerably, including both pediatric and adult patients as well as individuals with type 1 and type 2 diabetes mellitus, which differ in age of onset, disease duration, and associated systemic risk factors.

Systemic variables such as glycemic control, hypertension, and diabetes duration may further influence retinal microvascular alterations and act as potential confounders.

Another important limitation is that most available studies are cross-sectional in design, which limits the ability to determine causal relationships or the predictive value of OCTA-derived biomarkers for the development of diabetic retinopathy.

Furthermore, due to the narrative nature of this review and the heterogeneity of included studies, a formal risk-of-bias assessment was not performed. Future well-designed prospective longitudinal studies are therefore needed to clarify the temporal relationship between early retinal microvascular alterations and the onset of clinically detectable diabetic retinopathy.

## 4. Conclusions

The collected data indicate that OCTA reveals retinal and choroidal microcirculatory changes at a stage preceding the clinical diagnosis of diabetic retinopathy. Particularly significant are the selective involvement of the deep capillary plexus, alterations within the FAZ, and early areas of non-perfusion. OCTA parameters show potential for identifying patients at high risk of disease progression, monitoring disease course, and—prospectively—personalizing screening and therapeutic strategies.

Patients in whom clinically visible retinopathy has not yet developed represent the greatest therapeutic opportunity for preserving vision. Therefore, the development of sensitive and predictive retinal measurement methods capable of identifying individuals at risk of developing retinal changes would be highly valuable.

Early changes in diabetic retinopathy respond better to treatment; thus, identifying high-risk groups based on noninvasive OCTA examinations would be particularly beneficial. Considering the increasing incidence and prevalence of diabetes mellitus, opportunities for primary and secondary prevention are becoming increasingly limited. In many aspects of life, including healthcare, the use of artificial intelligence is becoming increasingly necessary.

Given the capabilities of OCTA and its noninvasive nature, it is anticipated that appropriate algorithms will soon enable faster and more accurate identification of patients with diabetes mellitus who are at risk of developing diabetic retinopathy, allowing timely implementation of appropriate treatment strategies.

Much work still lies ahead. We hope that the time required to identify the most sensitive biomarker and implement it into daily clinical practice will arrive sooner than expected.

## Figures and Tables

**Table 1:** Key OCTA-derived biomarkers associated with preclinical diabetic retinopathy and their clinical significance.

Category	OCTA Biomarker	Retinal Layer/Area	Reported Change in Preclinical DR	Clinical Significance	Key References
**Density-Based Metrics**	Vessel density (VD)	SCP	Decreased or unchanged	Early perfusion abnormalities	[5,6,7,9,15]
	Vessel density (VD)	DCP	Decreased	One of the most consistent early markers of retinal microvascular impairment	[4,9,15,27,28]
	Perfusion density (PD)	SCP/DCP	Decreased	Reduced retinal perfusion indicating early microvascular dysfunction	[15,27,28,29]
	Perfused capillary density (PCD)	SCP/DCP	Decreased	Sensitive indicator of capillary dropout and early ischemia	[9,15,28,29,30]
**FAZ-Related Parameters**	FAZ area	SCP/DCP	Increased or unchanged	Early ischemic alteration with considerable interstudy variability	[6,7,8,15,17,26,28,31,32]
	FAZ circularity/acircularity index	SCP/DCP	Decreased circularity/increased acircularity	Marker of early FAZ remodeling	[33,34,35]
	Peri-FAZ vessel density	SCP/DCP	Decreased	May precede overt enlargement of the FAZ	[23,27]
**Ischemia and Flow Impairment**	Capillary non-perfusion area	SCP/DCP	Increased	Indicator of retinal ischemia and microvascular damage	[27,36,37]
	Choriocapillaris flow deficit	CC	Increased	Possible indicator of early choroidal microvascular involvement	[15,21,22]
	Microaneurysm detection	SCP/DCP	Increased	Marker of early vascular pathology and disease activity	[34,38]
**Vascular Geometry and Network Complexity**	Choriocapillaris flow deficit (FD%)	CC	Increased	Possible indicator of early choroidal microvascular involvement	[15,21,22,32]
	Fractal dimension (FD)	SCP/DCP	Decreased	Reduced complexity of the vascular network	[29,30,39]
	Lacunarity	SCP/DCP	Increased network heterogeneity	Indicator of microcirculatory instability	[39]

VD—Vessel Density, PD—Perfusion Density, PCD—Perfused Capillary Density, FAZ—Foveal Avascular Zone, SCP—Superficial Capillary Plexus, DCP—Deep Capillary Plexus, and CC—Choriocapillaris.

**Table 2:** OCTA studies on patients with diabetes without clinical diabetic retinopathy.

Study/Year	Diabetes Type	Population	N (DM)	Control Group (n)	OCTA Parameters	Most Affected Layer	Main Findings
Dimitrova et al., 2017 [17]	T2DM	Adults	29	33	VD, FAZ	SCP, DCP	↑ FAZ ↓ VD
Carnevali et al., 2017 [10]	T1DM	Adults	25	25	VD, CC	DCP	↓ VD in DCP
Simonett et al., 2017 [57]	T1DM	Adults	28	23	VD, FAZ	DCP	↓ VD in DCP
Vujosevic et al., 2019 [16]	T1DM/T2DM	Adults	60	30	VD, GCL	SCP, DCP	↑ FAZ ↓ GCL thickness
Cao et al., 2018 [4]	T2DM	Adults	71	67	VD, FAZ	SCP, DCP, CC	↓ VD in SCP/DCP
Sousa et al., 2020 [53]	T1DM	Adults	24	24	VD, FAZ	SCP, DCP	↓ VD in SCP/DCP
Yang et al., 2020 [9]	T1DM/T2DM	Adults	92	92	VD, FAZ	SCP, DCP	↓ VD in SCP
Sacconi et al., 2024 [22]	T1DM	Adults	21	21	VD, VLD	DCP	↑ VD in DCP
Niestrata-Ortiz et al., 2019 [7]	T1DM	Children	112	30	FAZ	DCP	↑ FAZ
Gołębiewska et al., 2017 [6]	T1DM	Children	94	36	FD, FAZ	SCP, DCP	No difference
Koca et al., 2022 [20]	T1DM	Children	46	46	VD	SCP, DCP	↓ VD in SCP/DCP

VD—Vessel Density, FAZ—Foveal Avascular Zone, SCP—Superficial Capillary Plexus, DCP—Deep Capillary Plexus, CC—Choriocapillaris, T1DM—Type 1 diabetes mellitus, and T2DM—Type 2 diabetes mellitus, ↑ increase, ↓ decrease.

## Data Availability

No new data were created or analyzed in this study.

## Abbreviations

The following abbreviations are used in this manuscript:

CC	Choriocapillaris
DCP	Deep capillary plexus
DME	Diabetic macular edema
DR	Diabetic retinopathy
FAZ	Foveal avascular zone
FD	Fractal dimension
FD%	Choriocapillaris flow deficit
GCIPL	Ganglion cell–inner plexiform layer
ICP	Intermediate capillary plexus
NPDR	Non-proliferative diabetic retinopathy
OCTA	Optical Coherence Tomography Angiography
PCD	Perfused capillary density
PD	Perfusion density
RNFL	Retinal nerve fiber layer
SCP	Superficial capillary plexus
T1DM	Type 1 diabetes mellitus
T2DM	Type 2 diabetes mellitus
VD	Vessel density
VEGF	Vascular endothelial growth factor
VLD	Vessel length density
